# Administration of a selective retinoic acid receptor-γ agonist improves neuromuscular strength in a rodent model of volumetric muscle loss

**DOI:** 10.1186/s40634-021-00378-3

**Published:** 2021-08-12

**Authors:** Michael E. Whitely, Patrick B. Collins, Masahiro Iwamoto, Joseph C. Wenke

**Affiliations:** 1 Orthopaedic Trauma Department, United States Army Institute of Surgical Research, 3698 Chambers Pass, Building 3611, JBSA Fort Sam Houston, San Antonio, TX 78234 USA; 2 Department of Orthopaedics, University of Maryland School of Medicine, 655 W Baltimore St, Baltimore, MD 21201 USA

**Keywords:** Volumetric muscle loss, Muscle function, Palovarotene, RAR agonist

## Abstract

**Purpose:**

Volumetric muscle loss is a uniquely challenging pathology that results in irrecoverable functional deficits. Furthermore, a breakthrough drug or bioactive factor has yet to be established that adequately improves repair of these severe skeletal muscle injuries. This study sought to assess the ability of an orally administered selective retinoic acid receptor-γ agonist, palovarotene, to improve recovery of neuromuscular strength in a rat model of volumetric muscle loss.

**Methods:**

An irrecoverable, full thickness defect was created in the tibialis anterior muscle of Lewis rats and animals were survived for 4 weeks. Functional recovery of the tibialis anterior muscle was assessed in vivo via neural stimulation and determination of peak isometric torque. Histological staining was performed to qualitatively assess fibrous scarring of the defect site.

**Results:**

Treatment with the selective retinoic acid receptor-γ agonist, palovarotene, resulted in a 38% improvement of peak isometric torque in volumetric muscle loss affected limbs after 4 weeks of healing compared to untreated controls. Additionally, preliminary histological assessment suggests that oral administration of palovarotene reduced fibrous scarring at the defect site.

**Conclusions:**

These results highlight the potential role of selective retinoic acid receptor-γ agonists in the design of regenerative medicine platforms to maximize skeletal muscle healing. Additional studies are needed to further elucidate cellular responses, optimize therapeutic delivery, and characterize synergistic potential with adjunct therapies.

## Introduction

Skeletal muscle possesses a robust capacity to recover from injury, stemming from a population of resident progenitor cells who ultimately activate and fuse to repair damaged myofibers [10, 24, 50]. In cases of volumetric muscle loss (VML), however, wherein extensive portions of muscle tissue are lost, this healing cycle breaks down. The loss of progenitor cells and native extracellular matrix combine with an unrelenting immune response that promotes formation of nonfunctional fibrous tissue and severely inhibits de novo fiber regeneration [22, 32, 33]. To date, the clinical standard of care remains surgical placement of muscle flaps followed by extensive rehabilitation [8]. Promising experimental platforms, such as minced muscle graft transplantation, improve muscle function and fiber regeneration, but have been unable to fully mitigate the pathological response and suffer from finite availability [1]. Tissue engineering aims to overcome limited availability by combining more readily available biomaterial scaffolds, most often in the form of decellularized or polymeric matrices, with specific cell populations and bioactive cues to generate readily available tissue grafts [45]. The engineered grafts are then combined with rehabilitation regimens to introduce proper mechanical stimuli to the regenerating environment [13, 38, 39, 47]. The conversion of mechanical stimuli to biochemical and biomechanical signals via a series of mechanotransduction pathways is vital to directing satellite cell activation, muscle fiber hypertrophy, and extracellular matrix structure [16, 23, 34]. In contrast to other targets of tissue engineering, however, a breakthrough drug or bioactive factor has yet to be established that adequately promotes repair and regeneration of severe VML injuries. Therefore, development of a drug-based treatment that can be used in combination with current tissue engineering and regenerative rehabilitation programs to improve regenerative capacity and facilitate functional recovery would be invaluable.

Retinoic acid is vital to numerous developmental processes including skeletogenesis and myogenesis [30, 41]. Selective agonists of nuclear retinoic acid receptor-γ (RARγ) have emerged as a potential potent tool for treatment of skeletal disorders including hereditary multiple osteochondromas and fibrodysplasia ossificans progressiva, the latter being a debilitating condition involving pathological formation of bone in soft tissue sites [5, 18, 26, 43]. The mechanisms that provide the foundation for drug efficacy in these conditions including suppression of chondrocyte proliferation and matrix production have further extended to the proposed treatment of primary bone sarcomas, including chondrosarcoma, where these suppressive properties may aid in halting tumor growth and improving efficacy of chemotherapies [42]. Recently, it was observed that RARγ agonists may provide additional benefit to muscle regeneration and reduce the deposition of fibrous and adipose tissues in muscle defects [11]. From this, we hypothesized that the anti-fibrotic and pro-myogenic influences provided by these drugs may aid in altering the pathological environment observed in VML injuries, allowing for increased functional recovery and repair. This study was designed to determine the ability of the selective RARγ agonist, palovarotene (R667), to facilitate recovery of neuromuscular strength in vivo using a clinically relevant model of VML and further highlight its future potential in skeletal muscle repair.

## Methods

### Animals

Research was conducted in compliance with the Animal Welfare Act, the implementing Animal Welfare regulations, and the principles of the Guide for the Care and Use of Laboratory Animals, National Research Council. The facility’s Institutional Animal Care and Use Committee approved all research conducted in this study. The facility where this research was conducted is fully accredited by the AAALAC. Male Lewis rats (350–450 g; ~ 11 weeks of age) received pre-surgical administration of buprenorphine-SR (1.2 mg/kg; s.c., ~ 30 min prior) for pain management and were observed post-surgery for signs of distress and abnormal changes in mobility. Animals were euthanized under anesthesia after 4 weeks with a lethal dose of pentobarbital (Fatal Plus) and tissues harvested, Table 1.

**Table 1:** Gross anatomy and muscle weights

Experimental Group	Sample Size	Defect Weight [mg]	Endpoint Body Weight [g]	TA Weight [mg]	EDL Weight [mg]
**Sham**	6	–	367.2 ± 4.6	597.9 ± 18.9	163.1 ± 1.9
**No Repair**	6	71.5 ± 9.2	372.5 ± 9.3	566.9 ± 25.1	163.4 ± 9.6
**R667**	6	71.1 ± 10.5	355.8 ± 5.8	506.6 ± 42.4	170.4 ± 11.2

### VML model

A full thickness defect was created in the tibialis anterior (TA) muscle as previously reported [20, 21]. Briefly, an incision was made along the lateral aspect of the TA muscle under isoflurane anesthesia. Following blunt dissection of the skin and fascia, a metal plate was inserted between the TA muscle and the underlying extensor digitorum longus (EDL) muscle. The defect was created in the middle third of the TA using a 6 mm biopsy punch against the plate. The SHAM control group did not receive biopsy. Fascia and skin were closed with absorbable suture and skin clips.

### R667 administration

R667 (palovarotene, CAS410528–02-8) was administered (300 uL) at a concentration of 1 mg/kg via oral gavage three times per week beginning 1 week after surgery and was continued though the duration of the study. R667 solutions were prepared in dimethyl sulfoxide and mixed with corn oil at a ratio of 30:70 DMSO:corn oil. Vehicle control animals received an R667-absent solution of DMSO and corn oil. Treatment regimen was determined by scaling the effective dose range of 1.2–4.0 mg/kg established in previous preclinical mouse models of heterotopic ossification according to FDA issued guidance for industry [5, 11, 15, 43]. According to this guidance, 1.2–4.0 mg/kg palovarotene in mouse is roughly equivalent to 0.1–0.33 mg/kg in human and 0.5–2.0 mg/kg in rat.

### In vivo neuromuscular strength assessment

Muscle Function was assessed using a dual-mode lever system (Aurora Scientific, Inc.; Aurora, Canada: Mod. 305b) [7, 21]. Anesthetized animals were placed in a supine position with knee and ankle joints fixed at right angles. The foot was fastened to a pedal coupled to a servomotor-controlled force-displacement transducer and needle electrodes inserted percutaneously around the peroneal nerve. Optimal voltage (3–9 V) was identified using a series of tetanic contractions (5–10 contractions, 150 Hz, 0.1 ms pulse width, 400 ms train). The distal tendon of the EDL muscle was severed to isolate force production. Force to torque conversion was performed using a standardized 3 mm moment arm, followed by normalization to body weight [21].

### Histological analysis

A section of the defect region was collected and fixed in buffered formalin. Specimens were embedded in paraffin, sectioned into 4-μm slices, and stained using standard protocols for Masson’s Trichrome. Images were acquired using Axio Scan.Z1 microscope and ZEN imaging software (Carl Zeiss Microscopy; Jena, Germany).

### Data analysis

Data is reported as the mean ± SEM. Statistical analysis was conducted using GraphPad Prism 7.01 (GraphPad Software Inc., La Jolla, CA). Statistical significance is defined as *p* < 0.05 using student t-test or one-way ANOVA with Tukey post-hoc tests for multiple comparisons where appropriate.

## Results

### In vivo neuromuscular strength assessment

**Fig. 1:**
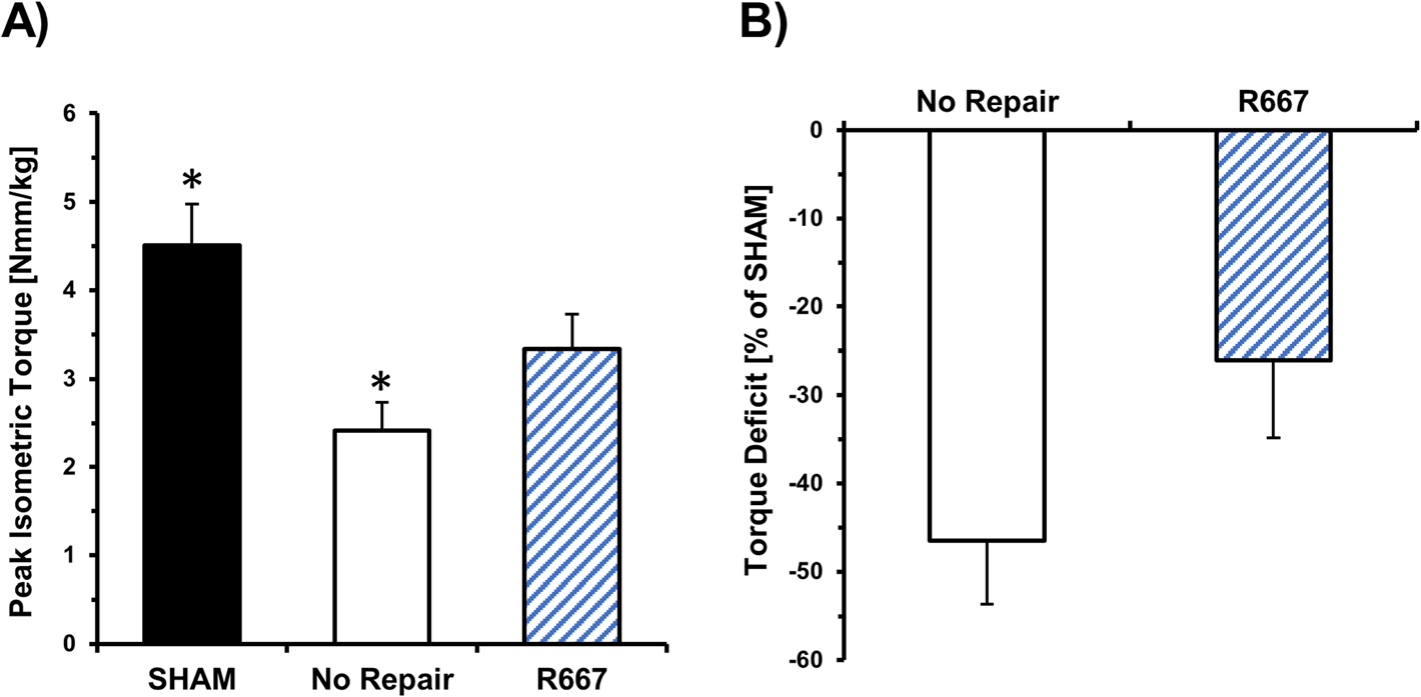
Oral administration of R667 improves neuromuscular function 4 weeks after volumetric muscle loss. Peak isometric torque normalized to body weight (A) and percent functional deficit relative to SHAM control (B) demonstrate improved functional capacity in the R667 treatment group relative to the No Repair group. *Indicates significant difference between SHAM and No Repair with *p* < 0.05. Values are presented as Mean ± SEM

Functional recovery of the tibialis anterior muscle was assessed in vivo via neural stimulation and determination of peak isometric torque 4 weeks after injury. VML affected limbs with No Repair exhibited maximum isometric torque production of 2.41 ± 0.32 Nmm/kg body weight and were significantly reduced compared to SHAM (*p* = 0.006), Fig. 1A. The 46% torque deficit in comparison to SHAM illustrates the lack of functional recovery that is a defining characteristic of VML injuries, Fig. 1B. In contrast, treatment with R667 increased torque production to 3.33 ± 0.39 Nmm/kg body weight and lowered the functional deficit compared to SHAM to 26%. This change in functional deficit for R667 treatment compared to No Repair, however, did not reach statistical significance (*p* = 0.099), Fig. 1B.

### Histological analysis

**Fig. 2:**
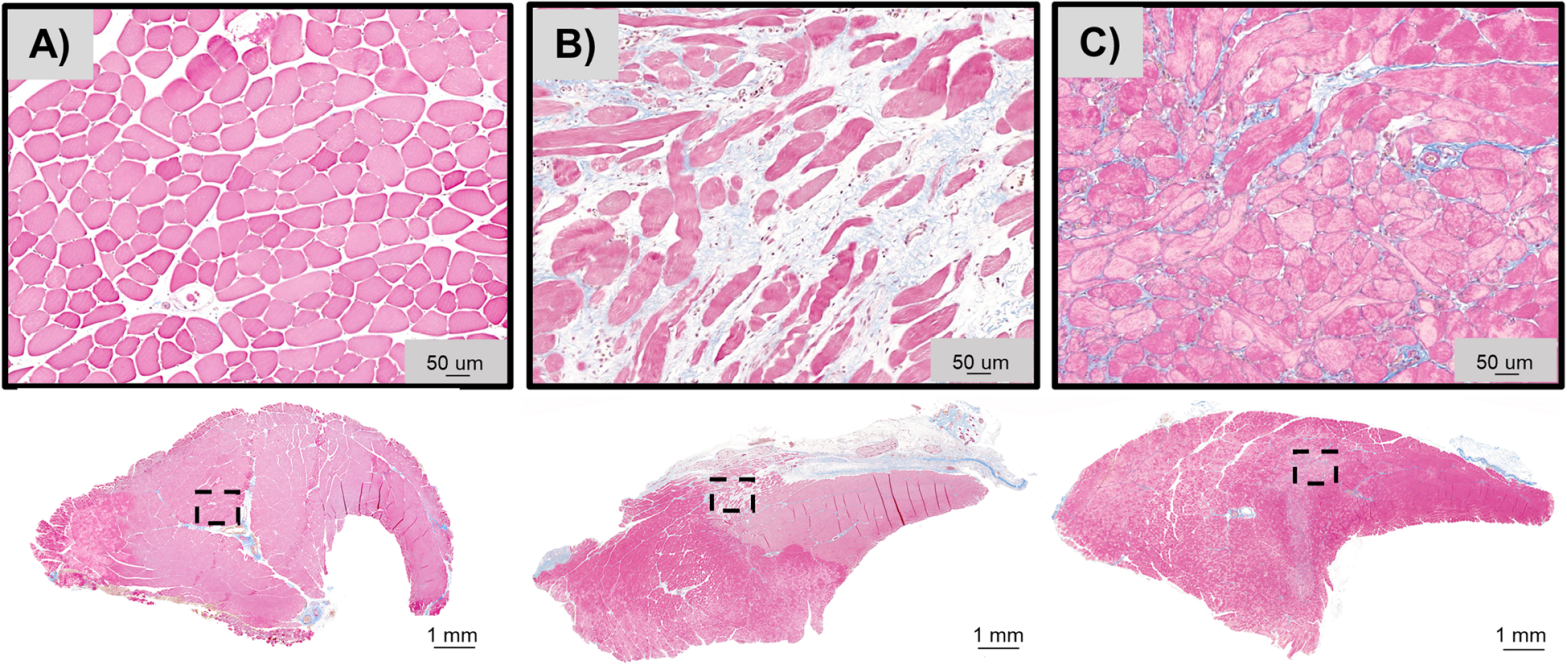
Histological analysis of tibialis anterior muscle 4 weeks post-injury. Masson’s Trichrome staining of SHAM (A), VML injured No Repair (B), and VML injured R667 treated (C) TA muscles. Muscle, fibrous, and adipose tissues are stained red, blue, and white, respectively. Scale bars are 1 mm for whole mount images and 50 μm for regions of interest

Histological staining was performed with Masson’s Trichrome to qualitatively assess the level of fibrous tissue deposition into the defect site following injury. As expected, excessive fibrotic scarring was observed following VML injury with the No Repair group consisting of sparsely populated myofibers surrounded by elevated amounts of extracellular matrix deposition, Fig. 2A & B. In contrast, a qualitative reduction in fibrous scarring was observed at the site of VML in R667 treated animals, Fig. 2C.

## Discussion

The primary aim of this study was to assess the ability of a selective RARγ agonist to improve neuromuscular strength in an irrecoverable, rat model of volumetric muscle loss that mimics the persistent functional deficits that present clinically. The results presented here are highly encouraging as they suggest that pharmacological activation of RARs may be a facile and promising method to improve functional recovery and repair of severe skeletal muscle injuries. RARγ agonist treatment benefits have previously demonstrated the ability to translate from preclinical models to patient use with phase 3 clinical investigation of fibrodysplasia ossificans progressive (NCT03312634) and phase 2 investigation of multiple osteochondromas (NCT03442985) currently ongoing [37]. A generally favorable safety profile has been observed with treatment strategies similar to those used here, with daily, 5 mg doses being well tolerated for greater than a year, and 20 mg doses routinely administered in response to disease flare ups [37, 44, 46] Side effects are consistent with general retinoid use with the most reported being mucocutaneous effects of the skin, eye, and gut. Adverse effects on skeletal growth in younger populations are being actively monitored, however, and future risk-benefit assessments will likely be needed in these populations [31, 37]. Of note, the level of functional recovery observed here is similar to that achieved with the tissue engineering-based strategy of minced muscle graft repair [7, 25]. It is hypothesized that combination of RARγ agonists with such cell- and/or scaffold-based systems may be a method to further improve their regenerative capacity and elevate muscle healing beyond current thresholds. Although the precise mechanism by which R667 facilitates restoration of muscle function is not fully elucidated in this study, key pro-myogenic and anti-fibrotic characteristics of retinoid signaling may provide insight and potential avenues for future exploration.

Retinoids exert transcriptional control through binding of nuclear retinoic acid receptors (α, β, and γ isoforms), followed by complexing with retinoid X receptors [12, 14]. These complexes bind to retinoid response elements of target genes and interact with a host of repression and activation factors to control expression patterns. In the absence of retinoid binding, unliganded complexes facilitate transcriptional repression in association with co-repressors such as nuclear receptor co-repressors 1 and 2. Co-repressor activity has been shown to influence muscle mass, oxidative metabolism, and exercise capacity in rodent models [48]. In a study highlighting the importance of γ isoform-specific RAR action in skeletal muscle repair, Di Rocco et al. identified dynamic temporal profiles in retinoid signaling following injury and observed that RARγ-null mice experienced marked delays in healing [11]. Furthermore, histological characterization of a cautery induced muscle defect revealed that orally administered R667 improved new myofiber formation and reduced the deposition of fibrous and adipose tissue inside the defect. This mitigation of pathological fibrosis has profound implications in muscle healing has been attributed to a reduction in Smad phosphorylation/canonical BMP signaling and increased activity of antichondrogenic Wnt/β-catenin pathways [36, 43, 49].

The persistent functional deficits that accompany excessive fibrous tissue deposition in VML injury have been extensively reported [2, 9, 22]. Of note, however, is that a reduction in fibrous tissue deposition alone, without a simultaneous improvement in fiber repair and regeneration, is detrimental to muscle function as it removes a potential conduit for force transmission across the defect [19]. This suggests that improved myofiber regeneration is likely occurring in conjunction with a reduction of fibrous scarring in R667 treated animals. In support, the referenced pathways influenced by retinoid signaling hold significant and complex roles in directing muscle progenitor activity. Wnt/β-catenin signaling, for example, has been implicated in promotion of satellite cell self-renewal, as well as in actively driving myogenic differentiation [4, 28, 35, 40]. Furthermore, the modulation of canonical BMP and adipogenic signaling in response to increased retinoid activity could potentially be removing significant barriers to myogenesis that present after injury [3, 17].

A noted limitation of the presented work is the absence of quantitative histological assessment of fibrous tissue deposition at the VML site. Future studies will need to evaluate collagen deposition and organization, progenitor cell activity, and myofiber regeneration to provide a better mechanistic understanding of improvements in functional recovery. It should also be noted that this work focused on recovery of muscle function in young rats. Multiple studies have reported on the deleterious effects of age on satellite cell activity and demonstrated the reduced response of aged rats to regenerative therapies for VML repair [6, 27, 29]. Additional study will be needed to identify age related effects on RARγ agonist-induced muscle healing.

## Conclusions

This study serves as an additional data point to suggest that selective RARγ agonists may hold promise in the treatment of severe skeletal muscle injury and warrant further investigation. Here, we demonstrate that oral administration of R667 was able to increase neuromuscular strength in a rat model of volumetric muscle loss. Additional studies are needed to further elucidate RARγ-induced cellular responses and characterize synergistic potential with adjunct therapies. Ultimately, this drug may prove a useful tool in the design of novel treatment paradigms to maximize healing of skeletal muscle.

## Data Availability

The datasets used and/or analyzed during the current study are primarily presented in the current manuscript and are available from the corresponding author on reasonable request.

## Abbreviations

VML: Volumetric muscle loss
RAR: Retinoic acid receptor
R667: Palovarotene
TA: Tibialis anterior
EDL: Extensor digitorum longus
